# Recombinants Are the Key Drivers of Recent PRRSV-2 Evolution

**DOI:** 10.3390/pathogens14080743

**Published:** 2025-07-29

**Authors:** Clarissa Pellegrini Ferreira, Lucina Galina-Pantoja, Mark Wagner, Declan C. Schroeder

**Affiliations:** 1 Department of Veterinary Population Medicine, College of Veterinary Medicine, University of Minnesota, 1365, Gortner Ave, Falcon Heights, MN 55108, USA; cpellegr@umn.edu; 2 Genus plc PIC, Hendersonville, TN 37075, USA; lucina.galina@genusplc.com; 3 Fairmont Veterinary Clinic, Fairmont, MN 56031, USA; mwagner@fmtvets.com

**Keywords:** PRRSV, whole-genome sequencing, recombination, phylogenetics, ORF5, viral evolution, swine, lineage classification

## Abstract

Porcine reproductive and respiratory syndrome virus remains one of the most economically significant pathogens in swine production, with PRRSV-2 being the dominant variant in the United States. While lineage classification has traditionally relied on ORF5 sequencing, recent studies suggest that this single-gene approach may overlook key evolutionary events such as recombination. In this study, we performed whole-genome sequencing and phylogenetic analysis of seven PRRSV-2 isolates collected in the U.S. between 2006 and 2024. Using reference-guided assembly, lineage assignment, and recombination detection with RDP5 and SIMplot, we identified discordant phylogenetic placements between ORF5 and whole genomes in four of the seven isolates. These discordances were explained by multiple recombination events affecting different genomic regions, particularly ORF2–ORF7. In contrast, three isolates showed phylogenetic concordance and no strong evidence of recombination. Our findings demonstrate that recombination plays a significant role in shaping PRRSV-2 evolution and highlight the limitations of ORF5-based lineage classification. Whole-genome surveillance is therefore essential to accurately track viral diversity, detect recombinant strains, and inform control strategies. This work underscores the need for a broader adoption of full-genome analysis in routine PRRSV surveillance and research.

## 1. Introduction

Porcine reproductive and respiratory syndrome virus (PRRSV) is a major pathogen in the swine industry, causing respiratory disease, disrupting reproduction, and increasing mortality in growing pigs, which reduces production efficiency and contributes to significant economic losses worldwide [1]. In the U.S., most outbreaks are attributed to PRRSV-2, which is caused by *Betaarterivirus suid 2*, an enveloped RNA virus from the Arteriviridae family within the Nidovirales order [2].

PRRSV-2 exhibits considerable genetic variability, with virulence differing between strains. This variability is primarily due to its high mutation rate, a common trait among RNA viruses, which lack RNA proofreading during replication [3]. Additionally, recombination plays a significant role in the genetic diversity and evolution of PRRSV-2, facilitating the exchange of genomic segments between co-infecting viral variants [4,5]. This process can lead to the emergence of novel variants with significant implications for epidemiology and immune response.

Genetic diversity in PRRSV-2 is not evenly distributed across the genome. While some regions remain highly conserved, others—such as the segments of ORF1a encoding nonstructural proteins (NSP1 and NSP2) and structural genes in ORF3–5—exhibit significant variation [3]. This variation results from a combination of mutations and recombination events, potentially leading to mosaic viruses that challenge lineage classification [6]. ORF5, in particular, is a key target for PRRSV classification due to its role in encoding the major envelope glycoprotein 5 (GP5), which is critical for viral entry and immune recognition [4,5]. Historically, PRRSV-2 was classified using the restriction fragment length polymorphism (RFLP) analysis of ORF5; however, phylogenetic classification based on whole-genome sequencing has since provided a different view of PRRSV evolution [7].

In this study, we analyze seven PRRSV-2 isolates collected from various U.S. locations between 2006 and 2024 to assess lineage stability and any evidence to support an alternative evolutionary event that could explain its emergence. Using complete genome vs. ORF5 sequence comparisons, we exploit phylogenetic inference relationships to affirm that ongoing surveillance efforts must include PRRSV-2 whole-genome sequence analysis.

## 2. Materials and Methods

### 2.1. PRRSV Isolates

Isolate KS2006-72109 L6 was sourced from Dr. Raymond Rowland, University of Illinois, Department of Pathobiology, 3205 Country Bend Lane, Champaign, IL; isolate 1-4-4 L1C UIL21-012 was sourced from Dr. Ying Farm, University of Illinois, Department of Pathobiology, 2001 South Lincoln Avenue M/C 002, Urbana, IL; isolates USA-NE-26342-1 (L1H), USA-OK-27915-12 (L1E), USA-IN-65239-GA (L1A), and USA-IL-23295-GA (L5A) were sourced from Dr. Jianqiang, Department of Veterinary Diagnostic and Production Animal Medicine, Iowa State University, 1577 Vet Med, 1800 Christensen Dr, Ames, IA. Isolate USA-MN-24-00737-0 was collected by Dr Mark Wagner from serum taken from unthrifty nursing piglets during an acute clinical break in a previously PRRS-positive herd.

### 2.2. RNA Extraction and cDNA Synthesis

Total RNA from seven PRRSV-2 isolates (Table 1) was extracted from 200 μL of serum collected on day 7 using the NucleoMag^®^ Virus kit (TaKaRa Bio USA, Inc., San Jose, CA, USA) with the KingFisher Flex Magnetic Particle Processor (Thermo Fisher Scientific, Waltham, MA, USA), following the manufacturer’s protocol.

For first-strand cDNA synthesis (adapted from Schroeder et al., 2021 [8], 4 μL of extracted RNA was mixed with 1 μL of a 10 μM pooled primer mix (Table A1) and 1 μL of 10 mM dNTPs. After incubation at 70 °C for 5 min and cooling to 4 °C, 2.5 μL of Template Switching Buffer, 0.5 μL of 75 μM TSO Oligonucleotide (sequence: AAGCAGTGGTATCAACGCAGAGTACrGrGrG), and 1 μL of Template Switching Enzyme were added. Synthesis was performed at 42 °C for 90 min and terminated at 85 °C for 5 min. The reaction was then held at 4 °C until further processing.

Second-strand cDNA was synthesized in a 25 μL reaction, including 2 μL of first-strand product, 0.25 μL of PrimeSTAR HS Polymerase, 5 μL of 5X PrimeSTAR buffer, 2 μL of 2.5 mM dNTPs, 1 μL of 10 μM TSO primer (sequence: AAGCAGTGGTATCAACGCAGAGTAC), and 14.75 μL of molecular-grade water. PCR conditions were as follows: 94 °C for 1 min, 30 cycles of 98 °C for 10 s, 60 °C for 15 s, and 68 °C for 2.5 min, with a final hold at 4 °C.

Purified cDNA was obtained using CleanNGS DNA & RNA Clean-Up Magnetic Beads at a 1:1 bead-to-sample ratio. After washing twice with 80% ethanol and air-drying, cDNA was eluted in 20 μL of molecular-grade water and quantified using the Qubit^®^ 1X dsDNA HS Assay on a Qubit^®^ 4.0 Fluorometer (Thermo Fisher Scientific, Waltham, MA, USA).

### 2.3. Library Preparation and Sequencing

Sequencing libraries were prepared using the Rapid Barcoding Sequencing Kit (SQK-RBK114.24) (Oxford Nanopore Technologies, Oxford, UK), following the manufacturer’s instructions. Libraries were loaded onto R.10 flow cells (FLO-MIN114) and sequenced on the GridION platform (Oxford Nanopore Technologies, Oxford, UK) for 24 h using default settings.

### 2.4. Assembly, Annotation, and Phylogenetic Analysis

Basecalling and demultiplexing were performed using Dorado v0.9.1 with the high-accuracy model. Reads were reference-assembled against a selected set of PRRSV2 genomes (GenBank accession numbers MN073153, KF724407, MZ423533, MZ423534, MZ423535, and MZ423536) using Minimap2 v2.24 in Geneious Prime 2025.0.2. The ‘map-ont’ preset was applied, allowing up to five secondary alignments per read with a minimum secondary-to-primary alignment score ratio of 0.8. Consensus sequences were generated using the highest quality threshold (60%) and a 65% threshold for sequences lacking base quality scores. Regions with no coverage were assigned ‘N’. Sanger heterozygotes were called when the variant frequency exceeded 50%; however, all consensus assemblies were manually inspected to resolve ambiguous bases, with special attention to regions of low coverage (<20×). Open reading frames (ORFs) were manually annotated by comparison with reference genomes to ensure accurate gene boundaries and coding sequence integrity.

ORF5 sequences were used to assign lineages, and full-length ORF1–ORF7 sequences were aligned with available PRRSV-2 genomes from NCBI using MAFFT v7.490 with the ‘Auto’ algorithm and default scoring parameters (200PAM/k = 2 scoring matrix; gap open penalty: 1.53; offset value: 0.123). Phylogenetic trees were constructed using FastTree v2.1.11 in Geneious Prime 2025.0.2, applying the Jukes–Cantor substitution model with 20 rate categories. Local support values were computed under the default maximum-likelihood framework. Final trees were rooted with PRRSV-1 sequences as outgroup taxa to improve lineage resolution.

The aligned sequences were analyzed using RDP5 software (v.5.64) to detect potential recombination events. The analysis employed seven different methods integrated within RDP5 [9]: RDP [10], GENECONV [11], MaxChi [12], BootScan [13], SiScan [14], Chimaera [15], and 3Seq [16]. A recombination event was considered valid if it was detected by at least five of the seven methods, ensuring a high confidence threshold to minimize false positives. The results from RDP5 were subsequently imported into R v 4.4.1 to generate SIMplot graphs, which illustrate pairwise identity patterns along the genome, highlighting regions of potential recombination. Lineage identification was confirmed using the ISU PRRSView web tool developed by Iowa State University Veterinary Diagnostic Laboratory [17].

## 3. Results

The samples analyzed in this study are PRRSV-2 isolates collected from various locations in the United States between 2006 and 2024. All samples are part of BioProject PRJNA1193630. Detailed metadata for the analyzed isolates, including accession numbers, BioSample identifiers, SRA accessions, isolate names, collection dates, and geographic origins, are provided in Table 1.

For each isolate (KS2006-72109, UIL21-0712, USA-IL-23295-GA, USA-IN-65239-GA, USA-NE-26342-1, USA-OK-27915-12, and USA-MN-24-00737-0), a range of 2 to 143 thousand reads was used to assemble near-complete genome sequences.

**Figure 1:**
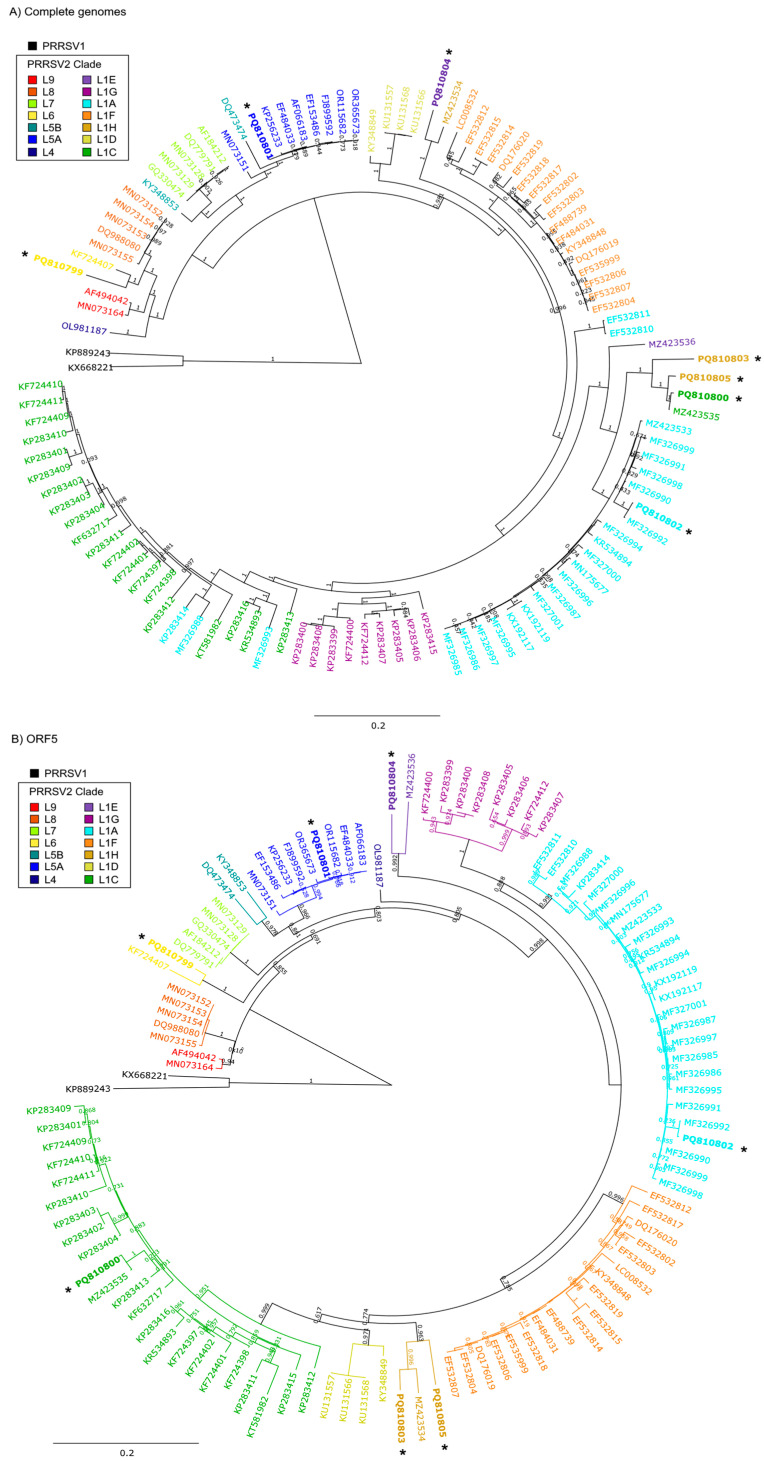
Phylogenetic nucleotide trees of the seven PRRSV isolates as they relate to PRRSV strains constructed from (**A**) genome sequences and (**B**) ORF5 sequences. The isolates (*) are labeled using their accession numbers, with the corresponding isolate names as follows: PQ810799 (KS2006-72109), PQ810800 (UIL21-0712), PQ810801 (USA-IL-23295-GA), PQ810802 (USA-IN-65239-GA), PQ810803 (USA-NE-26342-1), PQ810804 (USA-OK-27915-12), and PQ810805 (USA-MN-24-00737-0). The legend colors reflect the PRRSV lineages/sublineages, highlighting the genetic relationships and potential recombination events among the strains. ORF5 lineages/sublineages were assigned based on ORF5 phylogeny using the ISU PRRSView web tool [17]. The bar represents 2 mutations per 10 nucleotides, while the numbers at the branches in the tree are support values calculated by FastTree.

Phylogenetic inference trees were constructed from both the genome and ORF5 region of PRRSV-2 isolates as they relate to most strains reported in the U.S. since 1998 [18]. The genome-based tree (Figure 1A) provides a different view of evolutionary history based on the single gene ORF5 tree (Figure 1B), as highlighted by the color coding of four out of the seven isolates, which suggests that other regions of the PRRSV-2 genomes have a distinct evolutionary history from that of ORF5. Specifically, isolates UIL21-0712 (L1C), USA-NE-26342-1 (L1H), USA-OK-27915-12 (L1E), and USA-MN-24-00737-0 (L1H) appeared in different clades depending on whether the analysis was based on whole-genome sequences or ORF5. This disparity suggests that the ORF5 regions of these strains may have been acquired through recombination with other co-circulating co-infecting PRRSV variants.

The phylogenetic analysis also revealed that three isolates consistently cluster in the same clades in both the genome and ORF5-based trees. Notably, the isolates KS2006-72109 (L6), USA-IL-23295-GA (L5A), and USA-IN-65239-GA (L1A) exhibited congruent phylogenetic placements, suggesting that their ORF5 regions have evolved in a manner consistent with the rest of their genomic regions. This congruence implies that these isolates likely experienced a lineage-specific evolutionary trajectory based on specific localized mutation events, with minimal impact from large-scale changes such as rearrangement by recombination.

**Figure 2:**
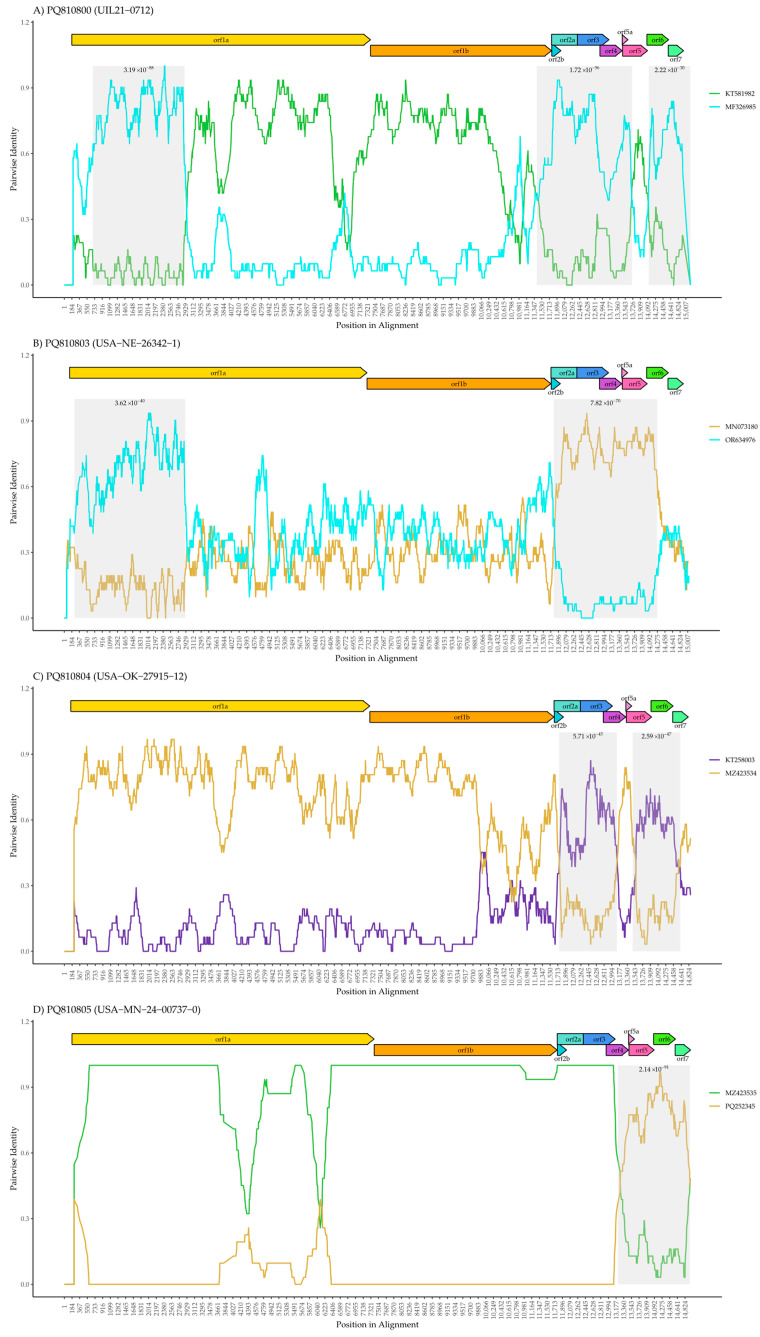
Recombination analysis based on RDP5 for four PRRSV isolates exhibiting discordant placements across the genome. The figure displays SimPlot-generated pairwise identity plots for four PRRSV strains: (**A**) UIL21-0712 (L1C), (**B**) USA-NE-26342-1 (L1H), (**C**) USA-OK-27915-12 (L1E), and (**D**) USA-MN-24-00737-0 (L1H). The *x*-axis represents the position in the genome’s alignment, while the *y*-axis shows the pairwise identity between the query strain and the contributing strains. Colored lines indicate the strains involved in the recombination events, with green representing L1C, blue representing L1A, gold representing L1H, and purple representing L1E. Shaded regions mark genomic areas where recombination breakpoints were detected, with associated *p*-values shown above the regions. The gene map at the top of each plot indicates the ORF structure of the viral genome.

To further explore the potential recombination events as suggested by the phylogeny for the four L1H, E, and C isolates, RDP5 clearly shows evidence of discordant placements between the genome and ORF5 gene, highlighting clear recombination breakpoints (Figure 2). In contrast, the recombination analysis of isolates KS2006-72109 (L6), USA-IL-23295-GA (L5A), and USA-IN-65239-GA (L1A) revealed no unified evidence of recombination breakpoints. For L6, two potential recombination events were detected using the Chimaera and GENECONV methods. However, the *p*-values associated with these events (3.98E-06 and 2.46E-02, respectively) were relatively high. Additionally, these events were not consistently detected across multiple methods, raising the possibility of false positives. Therefore, we conclude that no robust evidence of recombination was identified for these three isolates. The recombination analysis for the other four isolates, UIL21-0712 (L1C), USA-NE-26342-1 (L1H), USA-OK-27915-12 (L1E), and USA-MN-24-00737-0 (L1H), showed clear variations in pairwise identity along the genome, highlighting regions where significant changes occur (Figure 2).

The recombination analysis of UIL21-0712 identified contributions from both L1C and L1A (Figure 2A), which aligns with the genome phylogenetic analysis (Figure 1A). The presence of recombinant segments derived from both lineages elsewhere in the genome may have influenced the whole-genome phylogeny, leading to its clustering with L1A. However, the recombination plot shows that the ORF5 region is predominantly derived from L1C, which explains why it groups with this clade in the ORF5 phylogenetic analysis (Figure 1B). This observation highlights the importance of genome-based analysis to accurately capture the complex evolutionary history of PRRSV-2.

Concerning strain USA-NE-26342-1 (Figure 2B), two distinct regions exhibit marked changes in genetic similarity patterns. The first recombinant region, located within ORF1a, shows a higher similarity to OR634976 (L1A), while the second recombinant region, encompassing ORF2 to ORF6, displays increased similarity to MN073180 (L1H). The remaining genomic regions (outside of the primary recombinant segments) exhibit a range of similarity scores to both L1A and L1H, varying from 0.2 to 0.8. Notably, some segments reach a high similarity of 0.8 to L1A, indicating that specific regions of the genome share significant genetic identity with this lineage. This suggests that USA-NE-26342-1 may have acquired particular genomic fragments from an L1A-related strain through localized recombination events. The presence of both low and high similarity scores highlights the complex mosaic structure of the genome, where recombination did not uniformly impact all regions. Consequently, these high-similarity L1A segments may have influenced the whole-genome phylogeny, leading to its clustering with L1A.

USA-OK-27915-12 (Figure 2C) revealed a predominant genetic contribution from a strain such as MZ423534 (L1H) [8], as indicated by the consistently high pairwise identity throughout most of the genome. Two distinct recombinant regions were identified within ORF2 to ORF4 and ORF5 to ORF7, where the pairwise identity shifts to resemble strain KT258003 (L1E), as shown by the increased similarity of the purple line. These findings suggest that USA-OK-27915-12 primarily inherited its genome from L1H, with localized recombination events introducing segments from L1E in the ORF2-ORF4 and ORF5-ORF7 regions.

Figure 2D shows the recombination analysis of USA-MN-24-00737-0, revealing that the majority of the genome is derived from a strain such as MZ423535 or a related virus. MZ423535 itself is known to be a recombinant [8], suggesting that USA-MN-24-00737-0 inherited a complex recombinant background. However, a distinct recombinant region was identified from ORF4 to ORF7, where the pairwise identity shifts to resemble PQ252345 (L1H.18), shown by the increased similarity of the gold line. This indicates that USA-MN-24-00737-0 likely acquired the ORF4-ORF7 region from PQ252345, adding another layer of recombination to its genome. These results highlight the complex mosaic nature of this isolate, demonstrating how PRRSV-2 genomes can accumulate multiple recombination events over time.

To provide additional context, lineage assignments for these samples were compared with data from ISU PRRSView [17], a web-based tool that tracks PRRSV-2 variant trends in the U.S. This analysis confirmed that KS2006-72109 (L6) belongs to the 6.1 sublineage, which was last detected in 2022 and is now considered rare or extinct, indicating that it is no longer actively circulating. In contrast, USA-IN-65239-GA (L1A) was classified as 1A-unclassified, suggesting that while it remains within the broader L1A lineage, it does not fit into a well-defined active sublineage. Among the more recently collected isolates, UIL21-0712 (L1C) and USA-IL-23295-GA (L5A) were assigned to 1C.5 and 5A.1, respectively—both are classified as stable incidence, meaning that they continue to circulate at relatively steady levels. Meanwhile, USA-MN-24-00737-0 (L1H) belongs to 1H.18, an accelerating sublineage that has increased in frequency over the past year. In contrast, both USA-NE-26342-1 (L1H) and USA-OK-27915-12 (L1E) are part of 1H.9 and 1E.6, respectively—two decelerating sublineages that have been declining in detection over the past two years.

## 4. Discussion

The present study analyzed seven PRRSV-2 isolates collected from various locations in the United States between 2006 and 2024, revealing the complex evolutionary dynamics of these viruses. Through comprehensive phylogenetic and recombination analyses, we identified evidence of both lineage stability and recombination in several strains. Recombination in RNA viruses occurs when two or more viruses co-infect the same host–cell and segments from the viral genomes are exchanged during replication, resulting in recombinant genomes [19,20]. This process is a key mechanism driving genetic shifts in RNA viruses, enabling the rapid emergence of new variants with altered tropism, antigen profiles, and pathogenicity [19].

Notably, four of the five recent L1 isolates analyzed in this study likely originated from such co-infection events, highlighting the ongoing role of recombination in shaping the evolutionary trajectory of PRRSV-2. This phenomenon is not unique to the present study, as recombinants between PRRSV-2 lineages have been widely documented worldwide [3,6,20,21,22,23,24]. For instance, we previously analyzed PRRSV-2 variants from four distinct disease outbreaks at U.S. swine farms and found that the 2020 PRRSV-2 genome, classified as the L1C ORF5 sublineage, did not cluster with other L1C strains in the whole-genome phylogeny [8]. Instead, it grouped with L1A strains, indicating that the genetic relationships inferred from the complete genome differ significantly from those inferred from ORF5 alone. Similarly, it was reported that the L1C 2020–21 epidemic variant in the U.S. originated from a recombinant ancestor, where most genomic regions, including ORF5, were classified as L1C, while the ORF1a region showed closer similarity to L1A, highlighting recombination within two co-circulating L1 sub-lineages [7]. Our findings align with these previous observations [7,8], as both our phylogenetic and recombination analyses indicate that the four isolates likely originated from co-infections involving co-circulating PRRSV-2 strains.

The detection of recombination within PRRSV-2 lineages underscores the importance of understanding how genetic exchange can shape viral evolution. Notably, similar recombination events have been documented in other RNA viruses affecting swine and other mammals, such as the foot-and-mouth disease virus [25] and porcine epidemic diarrhea virus [26,27]. Recombination events between co-circulating strains in coronaviruses have been linked to the emergence of new variants, as seen in SARS-CoV-2 and its evolving lineages [28,29,30]. In influenza A (IAV), reassortment between subgenomic segments from co-infecting strains can result in significant genetic shifts, leading to the emergence of novel variants with pandemic potential [19]. For instance, the 2009 H1N1 pandemic virus emerged from a reassortment between swine influenza viruses that themselves were products of multiple prior reassortments involving avian, human, and swine-adapted strains [31]. Recently, a study on swine demonstrated that individuals co-infected with H1N1 and H3N2 generated a large number of IAV reassortants with multiple distinct genotypes; however, vaccination reduced reassortment by limiting the duration of co-infection [32]. Despite vaccination reducing reassortment, both vaccinated and non-vaccinated pigs exhibited similar levels of mutation rates within individual coding regions [32]. While high mutation rates also contribute to viral diversity, recombination and reassortment allow for the rapid emergence of new viral lineages beyond what mutation alone can achieve.

Mechanisms such as recombination and reassortment enable viruses to undergo abrupt genetic shifts, often resulting in new phenotypes with altered pathogenicity, transmissibility, or immune escape potential. In contrast, single-nucleotide mutations typically drive gradual adaptation, allowing fine-tuned responses to selective pressures such as host immunity or antiviral treatments. While less dramatic, these point mutations can accumulate over time, modifying key proteins involved in host–virus interactions or immune recognition [33]. Both mechanisms play essential roles in viral evolution, but the emergence of mosaic genomes through recombination or reassortment provides a more immediate pathway to phenotypic novelty. This raises a critical question in PRRSV-2 evolution: is recombination now the predominant driver of lineage diversification in the U.S., surpassing the slower accumulation of mutations detectable in ORF5 alone?

The biological significance of these recombination events is multifaceted. By accelerating genetic exchange, recombination increases the likelihood of producing high-fitness variants that may evade host immunity or reduce vaccine effectiveness [7]; however, this does not necessarily imply an increase in virulence. Recombination between distinct PRRSV-2 lineages can nevertheless result in the emergence of novel variants with increased pathogenicity, as previously reported [34]. Furthermore, such recombination events may lead to variants with novel antigenic properties, potentially compromising the efficacy of vaccines that rely heavily on ORF5-based lineage classification. It is important to note, however, that the actual phenotypic consequences of the recombinant viruses identified in this study remain uncertain and should be investigated further through experimental studies and field surveillance.

Another important consideration is that our recombination analysis was performed using whole-genome sequences available in the NCBI database. Therefore, it is crucial to recognize some limitations when interpreting our analysis. One key consideration is that the NCBI database may not accurately reflect the full genetic diversity of the U.S. PRRSV-2 population. This is partly because whole-genome sequencing is not commonly utilized in routine disease surveillance, primarily due to cost and accessibility challenges, and when it is performed, it often focuses on viruses associated with unusual clinical presentations [7]. As a result, the genomes from the three isolates that we identified as non-recombinant may appear stable, but because of unavailable data, we could not fully capture the diversity of the co-circulating PRRSV-2 at that time.

Additionally, it is possible that rather than being recombinant themselves, these genomes may act as parental lineages to other recombinant events, which may be selected because they are more resilient or epidemiologically successful. It is likely that these recombinants can give rise to new viruses, confusing our current lineage classification based on ORF5 alone, particularly if the newly formed variants exhibit enhanced survival or transmission. The common occurrence of these circulation recombinants could further support this hypothesis.

## 5. Conclusions

This study provides further evidence that recombination is a key driver of PRRSV-2 diversity and evolution in the U.S., as four of the seven analyzed isolates showed clear phylogenetic discordance between the ORF5 gene and whole-genome sequences, supported by recombination detection tools. In contrast, three isolates displayed phylogenetic concordance and no robust recombination signals, indicating that both stability and mosaicism can coexist within co-circulating lineages in farms. These findings demonstrate that reliance on ORF5 alone may fail to capture the complex evolutionary dynamics of PRRSV-2, reinforcing the importance of adopting whole-genome sequencing in routine surveillance. Broader and more systematic genomic monitoring will be critical for improving lineage classification, tracking emerging recombinants, and informing effective swine health management strategies.

## Figures and Tables

**Table 1:** Metadata for PRRSV-2 strains analyzed in this study, including strain name, BioSample identifier, SRA accession, isolate, collection date, geographic origin, total number of reads mapped to the reference, and genome length.

Accession Number	BioSample	SRA Accession	Isolate	Collection Date	State	Reads Mapped (bp)	Genome Length (bp)
PQ810799	SAMN45150044	SRR31590011	KS2006-72109 1-3-4 L6	2006	Kansas	143,295	15,397
PQ810800	SAMN45150045	SRR31590010	UIL21-0712 1-4-4 L1C	2021	Illinois	60,642	15,119
PQ810801	SAMN45150046	SRR31590009	USA-IL-23295-GA 2-5-2 L5A	2022	Illinois	116,314	14,642
PQ810802	SAMN45150047	SRR31590008	USA-IN-65239-GA 1-7-4 L1A	2014	Indiana	93,025	15,059
PQ810803	SAMN45150048	SRR31590007	USA-NE-26342-1 1-8-4 L1H	2022	Nebraska	4365	15,067
PQ810804	SAMN45150049	SRR31590006	USA-OK-27915-12 1-4-2 L1E	2022	Oklahoma	6207	14,915
PQ810805	SAMN45150050	SRR31590005	USA-MN-24-00737-0 1-12-2 L1H	2024	Minnesota	2042	15,125

## Data Availability

The genome sequences for this project have been deposited in GenBank under the following accession numbers: PQ810799 (KS2006-72109 1-3-4 L6), PQ810800 (UIL21-0712 1-4-4 L1C), PQ810801 (USA-IL-23295-GA 2-5-2 L5A), PQ810802 (USA-IN-65239-GA 1-7-4 L1A), PQ810803 (USA-NE-26342-1 1-8-4 L1H), PQ810804 (USA-OK-27915-12 1-4-2 L1E), and PQ810805 (USA-MN-24-00737-0 1-12-2 L1H). The Oxford Nanopore Technology reads are available under BioProject accession number PRJNA1193630.

## Abbreviations

The following abbreviations are used in this manuscript:

FMDV	Foot-and-Mouth Disease Virus;
GP5	Glycoprotein 5;
IAV	Influenza A Virus;
ISU	Iowa State University;
MAFFT	Multiple Alignment using Fast Fourier Transform;
NCBI	National Center for Biotechnology Information;
NSP	Non-Structural Protein;
ORF	Open Reading Frame;
PCR	Polymerase Chain Reaction;
PEDV	Porcine Epidemic Diarrhea Virus;
PRRSV-2	Porcine Reproductive and Respiratory Syndrome Virus Type 2;
RDP5	Recombination Detection Program version 5;
RFLP	Restriction Fragment Length Polymorphism;
RNA	Ribonucleic Acid;
SARS-CoV-2	Severe Acute Respiratory Syndrome Coronavirus 2;
SRA	Sequence Read Archive;
TSO	Template Switching Oligonucleotide.

## Appendix A

**Table A1:** Primers used for the amplification of PRRSV-2 genome regions.

Binding Region	Direction	Sequence	Length	Final Conc. (µM)
3′UTR	Reverse	5′-CCCTAATTGAATAGGTGACTTAG-3′	23	10
ORF5	Reverse	5′-CGGANCCNTCNAGCACNACT-3′	20	10
ORF4	Reverse	5′-CCNTTTTCNCTCATNTCNG-3′	19	10
ORF2/ORF3	Reverse	5′-AGNTCGAANGANAANTTGCCCCT-3′	23	10
ORF1b	Reverse	5′-GTATCCGANTCNAACCCCCA-3′	20	10
ORF1b	Reverse	5′-GCCTGNNCAACCGTGCANTC-3′	20	10
ORF1b	Reverse	5′-ACNGGNGTNTGNAGCTCCTT-3′	20	10
ORF1b	Reverse	5′-GNCCACAGCGGGTCAAGCC-3′	19	10
ORF1a	Reverse	5′-GCNAANGCTTCAAGNTTNG-3′	19	10
ORF1a	Reverse	5′-GTCCANGNNTGNCCCATCAT-3′	20	10
ORF1a	Reverse	5′-ATCAANAAAAACACNANCCA-3′	20	10
ORF1a	Reverse	5′-ANACAAGANCCCCANCACTT-3’	20	10
ORF1a	Reverse	5’-GGNGGNGGNGTNTCGAGTATCA-3′	22	10
ORF1a	Reverse	5′-CCNGCNCCNTACCANTTG-3′	18	10

## References

[1] Fiers J., Cay A.B., Maes D., Tignon M. (2024). A comprehensive review on porcine reproductive and respiratory syndrome virus with emphasis on immunity. Vaccines.

[2] Kappes M.A., Faaberg K.S. (2015). PRRSV structure, replication and recombination: Origin of phenotype and genotype diversity. Virology.

[3] Martín-Valls G.E., Kvisgaard L.K., Tello M., Darwich L., Cortey M., Burgara-Estrella A.J., Hernández J., Larsen L.E., Mateu E. (2014). Analysis of ORF5 and full-length genome sequences of porcine reproductive and respiratory syndrome virus isolates of genotypes 1 and 2 retrieved worldwide provides evidence that recombination is a common phenomenon and may produce mosaic isolates. J. Virol..

[4] Paploski I.A.D., Pamornchainavakul N., Makau D.N., Rovira A., Corzo C.A., Schroeder D.C., Cheeran M.C.-J., Doeschl-Wilson A., Kao R.R., Lycett S. (2021). Phylogenetic structure and sequential dominance of sub-lineages of PRRSV type-2 lineage 1 in the United States. Vaccines.

[5] Yim-im W., Anderson T.K., Paploski I.A.D., VanderWaal K., Gauger P., Krueger K., Shi M., Main R., Zhang J. (2023). Refining PRRSV-2 genetic classification based on global ORF5 sequences and investigation of their geographic distributions and temporal changes. Microbiol. Spectr..

[6] Wang J., Lin S., Quan D., Wang H., Huang J., Wang Y., Ren T., Ouyang K., Chen Y., Huang W. (2020). Full genomic analysis of new variants of porcine reproductive and respiratory syndrome virus revealed multiple recombination events between different lineages and sublineages. Front. Vet. Sci..

[7] Pamornchainavakul N., Kikuti M., Paploski I.A.D., Makau D.N., Rovira A., Corzo C.A., VanderWaal K. (2022). Measuring how recombination reshapes the evolutionary history of PRRSV-2: A genome-based phylodynamic analysis of the emergence of a novel PRRSV-2 variant. Front. Vet. Sci..

[8] Schroeder D.C., Odogwu N.M., Kevill J., Yang M., Krishna V.D., Kikuti M., Pamornchainavakul N., Vilalta C., Sanhueza J., Corzo C.A. (2021). Phylogenetically distinct near-complete genome sequences of porcine reproductive and respiratory syndrome virus type 2 variants from four distinct disease outbreaks at U.S. swine farms over the past 6 years. Microbiol. Resour. Announc..

[9] Martin D.P., Varsani A., Roumagnac P., Botha G., Maslamoney S., Schwab T., Kelz Z., Kumar V., Murrell B. (2021). RDP5: A computer program for analyzing recombination in, and removing signals of recombination from, nucleotide sequence datasets. Virus Evol..

[10] Martin D., Rybicki E. (2000). RDP: Detection of recombination amongst aligned sequences. Bioinformatics.

[11] Padidam M., Sawyer S., Fauquet C.M. (1999). Possible emergence of new geminiviruses by frequent recombination. Virology.

[12] Smith J.M. (1992). Analyzing the mosaic structure of genes. J. Mol. Evol..

[13] Martin D.P., Posada D., Crandall K.A., Williamson C. (2005). A modified bootscan algorithm for automated identification of recombinant sequences and recombination breakpoints. AIDS Res. Hum. Retroviruses.

[14] Gibbs M.J., Armstrong J.S., Gibbs A.J. (2000). Sister-scanning: A Monte Carlo procedure for assessing signals in recombinant sequences. Bioinformatics.

[15] Posada D., Crandall K.A. (2001). Evaluation of methods for detecting recombination from DNA sequences: Computer simulations. Proc. Natl. Acad. Sci. USA.

[16] Lam H.M., Ratmann O., Boni M.F. (2018). Improved algorithmic complexity for the 3SEQ recombination detection algorithm. Mol. Biol. Evol..

[17] Iowa State University Veterinary Diagnostic Laboratory ISU PRRSView Web Tool. https://prrsv.vdl.iastate.edu/.

[18] Wesley R.D., Mengeling W.L., Lager K.M., Clouser D.F., Landgraf J.G., Frey M.L. (1998). Differentiation of a porcine reproductive and respiratory syndrome virus vaccine strain from North American field strains by restriction fragment length polymorphism analysis of ORF 5. J. Vet. Diagn. Investig..

[19] Pérez-Losada M., Arenas M., Galán J.C., Palero F., González-Candelas F. (2015). Recombination in viruses: Mechanisms, methods of study, and evolutionary consequences. Infect. Genet. Evol..

[20] Cui X.-Y., Xia D.-S., Luo L.-Z., An T.-Q. (2024). Recombination of porcine reproductive and respiratory syndrome virus: Features, possible mechanisms, and future directions. Viruses.

[21] Zhao K., Ye C., Chang X.B., Jiang C.G., Wang S.J., Cai X.H., Tong G.Z., Tian Z.J., Shi M., An T.Q. (2015). Importation and recombination are responsible for the latest emergence of highly pathogenic porcine reproductive and respiratory syndrome virus in China. J. Virol..

[22] Wang A., Chen Q., Wang L., Madson D., Harmon K., Gauger P., Zhang J., Li G. (2019). Recombination between vaccine and field strains of porcine reproductive and respiratory syndrome virus. Emerg. Infect. Dis..

[23] Zhou L., Kang R., Yu J., Xie B., Chen C., Li X., Xie J., Ye Y., Xiao L., Zhang J. (2018). Genetic characterization and pathogenicity of a novel recombined porcine reproductive and respiratory syndrome virus 2 among NADC30-like, JXA1-like, and MLV-like strains. Viruses.

[24] Cui X., Xia D., Huang X., Sun Y., Shi M., Zhang J., Li G., Yang Y., Wang H., Cai X. (2022). Analysis of recombinant characteristics based on 949 PRRSV-2 genomic sequences obtained from 1991 to 2021 shows that viral multiplication ability contributes to dominant recombination. Microbiol. Spectr..

[25] Abd El Rahman S., Hoffmann B., Karam R., El-Beskawy M., Hamed M.F., Forth L.F., Höper D., Eschbaumer M. (2020). Sequence analysis of Egyptian foot-and-mouth disease virus field and vaccine strains: Intertypic recombination and evidence for accidental release of virulent virus. Viruses.

[26] Li D., Li Y., Liu Y., Chen Y., Jiao W., Feng H., Wei Q., Wang J., Zhang Y., Zhang G. (2021). Isolation and identification of a recombinant porcine epidemic diarrhea virus with a novel insertion in S1 domain. Front. Microbiol..

[27] Peng Q., Fu P., Zhou Y., Lang Y., Zhao S., Wen Y., Wang Y., Wu R., Zhao Q., Du S. (2024). Phylogenetic analysis of porcine epidemic diarrhea virus (PEDV) during 2020–2022 and isolation of a variant recombinant PEDV strain. Int. J. Mol. Sci..

[28] Focosi D., Maggi F. (2022). Recombination in coronaviruses, with a focus on SARS-CoV-2. Viruses.

[29] Patiño-Galindo J.Á., Filip I., Chowdhury R., Maranas C.D., Sorger P.K., AlQuraishi M., Rabadan R. (2021). Recombination and lineage-specific mutations linked to the emergence of SARS-CoV-2. Genome Med..

[30] Pipek O.A., Medgyes-Horváth A., Stéger J., Papp K., Visontai D., Koopmans M., Nieuwenhuijse D., Oude Munnink B.B., Csabai I., VEO Technical Working Group (2024). Systematic detection of co-infection and intra-host recombination in more than 2 million global SARS-CoV-2 samples. Nat. Commun..

[31] Zimmer S.M., Burke D.S. (2009). Historical perspective–emergence of influenza A (H1N1) viruses. N. Engl. J. Med..

[32] Li C., Culhane M.R., Schroeder D.C., Cheeran M.C.-J., Galina Pantoja L., Jansen M.L., Torremorell M. (2022). Vaccination decreases the risk of influenza A virus reassortment but not genetic variation in pigs. eLife.

[33] Perfumo C.J., Pereda A., Jongkaewwattana A., Chen Z., Perez D.R., Ma J. (2020). Editorial: Emerging swine viruses. Front. Vet. Sci..

[34] Wei C., Liu C., Chen G., Yang Y., Li J., Dan H., Dai A., Huang C., Luo M., Liu J. (2025). Genetic characterization and pathogenicity of two recombinant PRRSV-2 strains from lineages 1, 3, 5, and 8 emerged in China. BMC Vet. Res..

